# An Observational Study of Maternal and Perinatal Outcome in Preeclampsia Cases in a Tertiary Care Center

**DOI:** 10.7759/cureus.59352

**Published:** 2024-04-30

**Authors:** Minal A Kalambe, Neelu N Soni, Sara Ali, Nandkishor J Bankar

**Affiliations:** 1 Obstetrics and Gynaecology, Datta Meghe Medical College, Datta Meghe Institute of Higher Education and Research (DU), Wardha, IND; 2 Obstetrics and Gynaecology, Choithram Hospital and Research Center, Indore, IND; 3 Microbiology, Jawarhal Nehru Medical College, Datta Meghe Institute of Higher Education and Research (DU), Wardha, IND

**Keywords:** icu, disseminated intravascular coagulation (dic), pregnancy induced hypertension (pih), nicu., eclampsia, hellp syndrome, preeclampsia

## Abstract

Introduction: Preeclampsia, a hypertensive disorder in pregnancy, is a multisystem disease of unknown etiology and is associated with an increased risk of maternal mortality and morbidity. Infants from preeclampsia mothers have significantly higher incidence of prematurity, somatic growth retardation, thrombocytopenia, low birth weight, respiratory distress syndrome, and long duration of admission to neonatal intensive care (NICU).

Aims and objectives: This study was done to study the maternal mortality and morbidity and foetal outcome in pregnant women with severe preeclampsia.

Method: This observational study was done in the Department of Obstetrics and Gynaecology, of a tertiary care centre, from the period October 2015 to October 2017. Data was collected from all 130 women attending the antenatal clinic of tertiary care hospital and ward admission and all details such as demographic details, obstetrics examination, and all clinical findings were noted and from that made results.

Result: After applying inclusion and exclusion criteria all 130 women were observed in this study. Among 130 women 47 were diagnosed with preeclampsia. Mainly primigravida women were diagnosed with preeclampsia in the 21-25 years group. Among 47 preeclampsia women, 39 women had a BMI of 19-25 kg/m^2^. Thirty-two of 47 (68.09%) women were diagnosed with preeclampsia around 36-39 weeks. Among all preeclampsia, 28 women out of 47 (59.5%) women delivered babies vaginally, 18 of 47 (38.3%) women delivered through cesarean section, and one of 47 (2.13%) underwent preterm vaginal delivery. In preeclampsia, women's babies were delivered mostly (25/47, 53.19%) ≤2.5 kg weight and only one baby was shifted to NICU because of low birth weight. Preeclampsia increases maternal mortality and morbidity but in this study mortality was not done because our hospital is a tertiary care center with all ICU (intensive care unit) and NICU setup.

Conclusion: Preterm births and cesarean deliveries were the mild to severe outcomes that were noted. ICU and NICU hospitalizations as a result of severe complications place a heavy demand on medical facilities. There are firm guidelines for the management of pregnancy-induced hypertension and its complications. For appropriate management, there is careful consideration of various factors, and individual case studies are required.

## Introduction

Worldwide, preeclampsia (PE) represents a significant obstetric problem. In addition to being one of the main causes of preterm delivery, it is one of the main three variables that increase maternal and fetal morbidity and mortality [1]. Preeclampsia affects about 8% to 10% of all pregnancies in India. In primgravidae, the incidence is 10% and in multigravidae, it is 5%. A recent confidential investigation of maternal death in the UK found hypertensive disorder which is the second leading direct cause of maternal death [2]. Preeclampsia in pregnant women is characterized by high blood pressure, equivalent to or above 140/90 mmHg for both systolic and diastolic blood pressure, and urine containing albumin protein [3]. When kidney damage occurs, the albumin that usually appears in the blood leaks into the urine. Kidney injury is indicated by albumin levels greater than 30 mg/L and the simultaneous appearance of these two grave issues [4]. Preeclampsia is a hypertensive disorder of unknown etiology (cause). Not only is obesity (excess weight increase) related to preeclampsia, but it is also a primary cause of heart problems. In addition to the adverse effects on maternal health, such as diabetes, heart disease, and hypertension, obesity during pregnancy can also affect fetal health, including premature birth and aberrant growth. Preeclampsia risk factors may include maternal age, numerous pregnancies, kidney failure, and in vitro fertilization. In the study, the effect remained statistically significant after adjustment for possible confounding factors, such as age, parity as well as regularity and duration of the menstrual cycle (odds ratio of 0.77) [5].

Preeclampsia is estimated to occur in 5 to 7% of all pregnancies and is one of the leading causes of maternal morbidity. Annually, preeclampsia is responsible for over 70,000 maternal deaths and 500,000 fetal deaths worldwide occur in low- and middle-income countries [6]. Preeclampsia is associated with an increased risk of maternal mortality, maternal morbidities such as convulsions, abruption placenta, pulmonary edema, intracerebral bleeding, acute renal failure congestive cardiac failure, hemolysis, elevated liver enzymes and low platelets (HELLP) syndrome, and disseminated intravascular coagulation (DIC) [7]. Infants from preeclampsia mothers have significantly higher incidence of prematurity, somatic growth retardation, thrombocytopenia, low birth weight, respiratory distress syndrome, and long duration of admission to neonatal intensive care (NICU) [8]. In developing countries, women are at 14 times higher risk of dying from obstetric complications compared to developed countries. Preeclampsia highlights the importance of regular prenatal care and vigilant monitoring of blood pressure during pregnancy. Early detection and timely intervention are critical to preventing and managing this life-threatening condition and reducing its associated complications [9].

## Materials and methods

This observational study was done in the Department of Obstetrics and Gynecology at Choithram Hospital and Research Centre, Indore. After satisfying inclusion and exclusion criteria, 130 pregnant women attending antenatal clinics, both outpatient and ward admissions, were included in this study.

Inclusion criteria were all pregnant women attending the antenatal clinic, both outpatient and ward admissions; women of any gravidity, parity, socioeconomic status, educational status, and/or without any high-risk pregnancy such as preeclampsia and diabetes mellitus, their legally acceptable representative willing to provide voluntary written informed consent were included in the inclusion criteria.

Exclusion criteria were previous history of preeclampsia or eclampsia, diabetic mellitus, chronic hypertension or essential hypertension, thyrotoxicosis, renal disease, Rh incompatibility, positive lupus anticoagulant anti-cardiolipin antibodies, and women who do not give consent should be excluded from this study. The data from the women was collected using a predesigned questionnaire, and an observational data collection method was used.

Details of the pregnant woman were noted, such as name, age, present symptoms, last menstrual period, details of the history of the clinical presentation of the patient, obstetrical history, past history, and family history. All antenatal women were followed up during pregnancy and delivery, up to seven days after delivery, for the development of pregnancy-induced hypertension and perinatal outcome.

Ethical consideration 

The procedure was presented for approval before the ethical committee. The study was launched within the organization following the ethics committee's proper approval. A written consent for participation, given voluntarily and independently of other approvals obtained in accordance with institutional protocols for the management of high-risk cases, was also obtained from each woman, either through her legally recognized representatives or herself, before her inclusion in the study. On June 26, 2016, we acquired permission to examine these individuals' medical records and use the data for research purposes from the Choithram Hospital Research Centre's medical records department and ethics committee via letter no. EC/June/16/06.

## Results

A total of 130 women were included in our study. Among 130 women 47 were diagnosed with preeclampsia, and out of 47, 23 women were in the age group 21-25 years. In normotensive women out of 83, 44 (53.01%) were in the age group 21-25 years. Out of 44 women with preeclampsia, 33 (70.21%) women were primigravidae; in our study maximum primigravidae women were among the 21-25 age group diagnosed with preeclampsia (Table 1).

**Table 1:** Risk of preeclampsia in relation to age of patients P value < 0.05 means significant In this study, the P value = 0.051, so the study was not significant. x^2^ – chi square, df - degrees of freedom

Age	Preeclampsia		Total N (%)	Statistical analysis
	Present N (%)	Absent N (%)		
Up to 20 years	0 (0.00%)	10 (12.05%)	10 (7.69%)	X^2^ value 7.7609, df = 3, P value = 0.0512, not significant
21-25 years	23 (48.94%)	44 (53.01%)	67 (51.54%)	
26-30 years	19 (40.43%)	22 (26.51%)	41 (31.54%)	
31-35 years	5 (10.64%)	7 (8.43%)	12 (9.23%)	
Total	47 (100%)	83 (100%)	130 (100%)	

Among the 47 women with preeclampsia, 39 (82.98%) had a normal BMI range of 19-25 kg/m², while among normotensive women, 63 (75.90%) fell within the same BMI range. These findings suggest that preeclampsia is influenced by the woman's BMI (Table 2).

**Table 2:** Risk of preeclampsia with BMI If the P value < 0.05 then the study was significant In this study, the P value was 0.004, so the study was significant x^2^ – chi-square, df - degrees of freedom, BMI- Body Mass Index

BMI	Preeclampsia		Total N (%)	Statistical analysis
	Present N (%)	Absent N (%)		
≤18	0 (0.00%)	13 (15.66%)	13 (10.00%)	X^2 ^value 13.0088, Df = 3, P value = 0.0046, significant
19-25	39 (82.98%)	63 (75.90%)	102 (78.46%)	
26-30	6 (12.77%)	7 (8.43%)	12 (9.23%)	
>30	2 (4.26%)	0 (0.00%)	3 (2.31%)	
Total	47 (100%)	83 (100%)	130 (100%)	

In the group of women with preeclampsia, among the 47 cases, 32 (68.09%) were diagnosed with preeclampsia between the 36th and 39th weeks of pregnancy. This indicates a late diagnosis of preeclampsia during pregnancy (Table 3).

**Table 3:** Risk of preeclampsia with gestational age If the P value < 0.05, then the study was significant In this study, the P value was 0.1711, so the study was not significant x^2^ – chi-square, df - degrees of freedom

Gestational Weeks	Preeclampsia		Total N(%)	Statistical analysis
	Present N (%)	Absent N (%)		
≤30 weeks	3 (6.38%)	0 (0.00%)	3 (2.31%)	X^2 ^value 5.0099, df = 3, P value = 0.1711, not significant
31-35 weeks	7(14.89%)	0 (0.00%)	7 (5.38%)	
36-39weeks	32(68.09%)	73 (87.95%)	105 (80.77%)	
≥40 weeks	5(10.64%)	10 (12.05%)	15 (11.54%)	
Total	47 (100.00%)	83 (100.00%)	130 (100.00%)	

Among the women with preeclampsia, 28 (59.57%) underwent full-term vaginal delivery, 18 (38.30%) underwent lower segment cesarean section (LSCS), and one (2.13%) underwent preterm vaginal delivery, all based on the indication for cesarean section (Table 4).

**Table 4:** Risk of preeclampsia with mode of delivery If the P value < 0.05, then the study was significant In this study, the P value was 0.9121, so the study was not significant x^2 ^– chi-square, df - degrees of freedom, FTVD - full-term vaginal delivery, LSCS - lower segment cesarean section, PTVD - preterm vaginal delivery

Mode of delivery	Preeclampsia		Total N (%)	Statistical analysis
	Present N (%)	Absent N (%)		
FTVD	28 (59.57%)	49 (59.04%)	77 (59.23%)	X^2 ^value 0.1839, df = 2, P value = 0.9121, not significant
LSCS	18 (38.30%)	33 (39.76%)	51 (39.23%)	
PTVD	1 (2.13%)	1 (1.20%)	2 (1.54%)	
TOTAL	47 (100%)	83 (100%)	130 (100%)	

The most common indication for cesarean section among women with preeclampsia was abnormal Doppler (six cases, 12.77%) and fetal distress (nine cases, 19.15%), whereas among normotensive women, fetal distress (12 cases, 14.46%), scar tenderness (five cases, 6.02%), and prolonged second stage of labor (five cases, 6.02%) were observed. The majority of preeclampsia cases, particularly those with fetal distress, underwent cesarean section (Table 5).

**Table 5:** Distribution of women according to indication of caesarean section If the P value < 0.05, then the study was significant In this study, the P value was 0.579, so the study was not significant x^2 ^– chi-square, df - degrees of freedom, LSCS - lower segment cesarean section, PROM - patient-reported outcome measures

Causes of LSCS	Preeclampsia		Total N(%)	Statistical analysis
	Present N (%)	Absent N (%)		
Abnormal Doppler	6 (12.77%)	3 (3.61%)	9 (6.92%)	x^2 ^value 0.3144, df = 1, P value = 0.579, not significant
Breech	2 (4.26%)	2 (2.41%)	4 (3.08%)	
Fetal distress	9 (19.15%)	12(14.46%)	21(16.15%)	
Malpresentation	0 (0.00%)	2 (2.41%)	2 (1.54%)	
Normal	29 (61.70%)	50 (60.24%)	79 (60.77%)	
Prolong second stage	0 (0.00%)	5 (6.02%)	5 (3.85%)	
PROM	0 (0.00%)	4 (4.82%)	4 (3.08%)	
Scar tenderness	1 (2.13%)	5 (6.02%)	6 (4.62%)	
Total	47 (100%)	83 (100%)	130 (100%)	

Among the 47 women with preeclampsia, 25 (53.19%) had babies weighing ≤2.5 kg, while 22 (46.81%) had babies weighing >2.5 kg. This indicates that in preeclampsia, more women had babies weighing <2.5 kg compared to normotensive women. It suggests that the weight of the baby is independent of preeclampsia (Table 6).

**Table 6:** Risk of preeclampsia with birth weight of baby If the P value < 0.05, then the study was significant In this study, the P value was 0.107, so the study was not significant x^2 ^– chi-square, df - degrees of freedom

Weight of baby kg	Preeclampsia		Total N (%)	Statistical analysis
	Present N(%)	Absent N (%)		
≤2.5 kgs	25 (53.19%)	32 (38.55%)	57 (43.85%)	x^2 ^value 2.6112, df = 1, P value = 0.107, not significant
>2.5 kgs	22 (43.85%)	51 (61.45%)	73 (56.15%)	
Total	47	83	130	
	100.00%	100.00%	100.00%	
Odds Ratio (cross product)	0.5522			

In women with preeclampsia, 13 (27.66%) babies were admitted to the NICU, whereas in normotensive women, only one (1.20%) baby was admitted. This suggests a higher incidence of NICU admissions for babies born to women with preeclampsia. In this study neonatal deaths were 0% (Table 7). Only two studies are available in the literature, which have been summarized in Table 7, only two studies are available on preeclampsia outcomes considering inclusion criteria of NICU admitted cases.

**Table 7:** Foetal outcome in other studies NICU - neonatal intensive care unit

Studies	NICU (%)	With mother (%)	Neonatal death (%)
Naik et al. (2017) [10]	17.46%	80.96%	1.59%
Sumathi et al. (2016) [11]	16.7%	77.5%	5.8%
This study	10.77%	89.23%	0%

## Discussion

Preeclampsia, a complicated multiorgan clinical condition, continues to be the leading cause of morbidity and mortality in both mothers and newborns. It is still challenging to find the perfect diagnostic and prognostic test as a preventive intervention. In Latin America and the Caribbean, hypertensive disorders account for more than 25% of maternal deaths, whereas in Africa and Asia they contribute to 9% of deaths. Further, 16% of maternal deaths have been attributed to hypertensive disorders [3]. The average incidence of preeclampsia is between 5 and 15%. The causes of pregnancy-induced hypertension are multiple, and are ably supported by multiple guidelines, nevertheless there is uncertainty around management, making preeclampsia care difficult and contentious. Among 47 women with preeclampsia, 23 (48.94%) and in normotensive women, 44 (53.01%) were in the age group 21-25 years. In our study, in the 21-25 age group 48.9% of women with preeclampsia were present, which was found to be higher than in other studies such as Kaku et al. [12], Nandanwar et al. [13], Aggarawal et al. [14] as 46%, 46%, 43% were found to have preeclampsia in the 21-25 age group. Still, the Nanthini et al. study [15] was higher than our study, as 53% of women were found to have preeclampsia in the same age group.

In women with preeclampsia, 33 (70.21%) were primigravidae, and in normotensive women, 47 (56.63%) were primigravidae. The study by Nanthini et al. [15] and Naik et al. [10] was comparable to our study. In the Kaku et al. [12] study, preeclampsia appeared 30-34 weeks earlier than ours. In women with preeclampsia, among 47 women, 32 (68.09%) were diagnosed with preeclampsia between 36-39 weeks of pregnancy. In the study by Nanthini et al. [15], 64.3% were diagnosed with preeclampsia between 36 and 40 weeks, and in Naik et al. [10], 52.3% of the cases developed preeclampsia between 37 and 40 weeks, which was comparable to our study, but Kaku et al. [12] found that preeclampsia was seen in 30-34 weeks, which was found to be earlier than the findings of our study. In this study, among 47 women with preeclampsia, only 44 or 95.7% of women without preeclampsia and only 4.26% with severe preeclampsia, which was comparable to Nanthini et al. [15] and Naik et al. [10] (15.26%) higher incidence of women with severe preeclampsia than our study.

The study by Bhalerao et al. [16] had a higher (73.21%) vaginal delivery rate than our study, 28/43 (59.57%), and the study by Naik et al. [10] (68.2%) was comparable to our study. In our study, 38.30% of cesarean sections were performed in preeclampsia, and the most common indication of cesarean sections with preeclampsia was abnormal Doppler (12.77%) and fetal distress (19.15%). Sumathi et al. [11] found an almost identical indication for a LSCS with preeclampsia.

In this study, 25 babies had a weight ≤2.5 kg, which means that preeclampsia women had baby weights less than 2.5 kg than normotensive women and because of that more NICU admission in preeclampsia than normotensive women. Because of that, there were more admissions to NICU admissions in preeclampsia than in normotensive women. Maternal mortality and complications are related to the onset and severity of the disease that are related to other studies in our study; no maternal death could be attributed to the prompt diagnosis of the disease and the start of treatment in our study. This outcome was nearly identical to that of the study carried out in Enugu, Nigeria, which did not show maternal deaths [17].

Limitations

Because of the smaller sample size in this study, external validity is necessary to produce better results. External validity concerns the applicability of study results from the study's sample to the target population as a whole. The present research has a few drawbacks. Since this was a hospital-based study with a relatively small patient group, our results cannot be generalized to the entire population. Significant problems such as low birth weight, growth retardation, and respiratory tract syndrome were observed during the trial period, but no maternal deaths were reported. Elevation in blood pressure had a significant association with maternal and fetal outcomes. Furthermore, more studies in this area are needed to calculate the possible benefits for mothers and their unborn children of better diet and home surveillance for the management of preeclampsia both before and after delivery.

## Conclusions

This work provides important new information on the intricate dynamics of preeclampsia and how it affects neonatal and maternal outcomes. This study shows a strong correlation between preeclampsia and the demographic factors of the patient. Preterm births and cesarean deliveries have been reported as mild to severe results. ICU hospital stays as a result of severe complications places a heavy demand on healthcare services. The results highlight the need for further research and early medical intervention to improve outcomes for those affected by this potentially harmful pregnancy condition. This study is essential for physicians, decision-makers and scholars who want to improve preeclamptic mother and perinatal care.
